# Changes in Brain Function Networks in Patients With Amnestic Mild Cognitive Impairment: A Resting-State fMRI Study

**DOI:** 10.3389/fneur.2020.554032

**Published:** 2020-09-30

**Authors:** Xiaoling Li, Feng Wang, Xiaohui Liu, Danna Cao, Lina Cai, Xiaoxu Jiang, Xu Yang, Tiansong Yang, Tetsuya Asakawa

**Affiliations:** ^1^First Affiliated Hospital, Heilongjiang University of Chinese Medicine, Harbin, China; ^2^Division of CT and MRI, Heilongjiang University of Chinese Medicine, Harbin, China; ^3^Department of Neurosurgery, Hamamatsu University School of Medicine, Hamamatsu, Japan; ^4^Research Base of Traditional Chinese Medicine Syndrome, Fujian University of Traditional Chinese Medicine, Fuzhou, China

**Keywords:** resting-state functional magnetic resonance imaging, functional connectivity, default mode network, executive control network, salience network, amnestic mild cognitive impairment

## Abstract

Patients with amnestic mild cognitive impairment (aMCI) are at high risk of developing dementia. This study used resting-state functional magnetic resonance imaging (rs-fMRI) and an independent component analysis (ICA) approach to explore changes in functional connectivity (FC) in the default mode network (DMN), executive control network (ECN), and salience network (SN). Thirty patients with aMCI and 30 healthy controls (HCs) were enrolled. All the participants underwent an rs-fMRI scan. The brain FC in DMN, ECN, and SN was calculated using the ICA approach. We found that the FC of brain regions in DMN decreased significantly and that of brain regions in ECN increased, which was in accordance with the findings of previous studies on Alzheimer's disease (AD) and aMCI. We also found that the FC of brain regions in SN increased, which was different from the findings of previous studies on AD. The increase in FC in brain regions in SN might result from different pathophysiological states in AD and aMCI, indicating that a decrease in FC in SN does not occur in a person with aMCI. These results are consistent with those of previous studies using the voxel-mirrored homotopic connectivity approach and seed-based correlation analysis. We therefore considered that the decrease in FC in DMN and the increase in FC in ECN and SN might be peculiar patterns observed on the rs-fMRI of a person with aMCI. These findings may contribute to the development of imaging biomarkers for the diagnosis of aMCI.

## Introduction

Mild cognitive impairment (MCI) is a pathological state between normal cognition and dementia. MCI is associated with many neurological diseases such as neurodegenerative diseases, cerebral vascular diseases, and metabolic symptoms. MCI has slight impact on activities of daily living (ADL), but patients with MCI have a high risk of developing dementia, for which there is no effective treatment. The annual conversion rate from MCI to dementia is reportedly ~10–15%, which is 10 times that in the normal population (1). Thus, MCI has been regarded as a crucial stage in which the long-term outcome of dementia may be improved if early diagnosis and intervention are achieved (2). MCI is commonly classified into two subtypes: amnestic MCI (aMCI) and non-amnestic MCI (naMCI). aMCI manifests predominantly as memory loss and is more likely to progress to Alzheimer's disease (AD), whereas naMCI is closely associated with forms of dementia other than AD (3). Because AD is a leading neurodegenerative disease, the relationship between aMCI and AD and the early diagnosis and treatment of aMCI have garnered much attention from clinicians.

Great efforts have been made to explore diagnostic and screening tools for aMCI, such as psychological batteries (2, 4, 5), cerebrospinal fluid biomarkers (6), and molecular imaging approaches (7). These methods have their special strengths and limitations. So far, there is no gold standard tool for the early diagnosis of aMCI, but resting-state functional magnetic resonance imaging (rs-fMRI) is often used for this purpose. The functional changes observed on fMRI of patients with AD have been well-documented. Commonly, resting static networks (long-term networks) are constructed based on function connectivity (FC) on long or full-length fMRI time series. Three large-scale cognition-related networks, the default mode network (DMN), executive control network (ECN), and salience network (SN), are widely used to investigate brain changes in the cognitive impairment state. Joo et al. (8) reported evident abnormal connections in the brains of patients with AD, particularly in the default mode network (DMN), executive control network (ECN), and salience network (SN). DMN is a task-negative as well as the most documented network associated with AD (9). Royall et al. (10) found that DMN atrophy was closely associated with the severity of cognitive impairment in patients with AD. Li et al. (11) found that the disruption of DMN was frequency dependent. Banks et al. (12) found that left lateralization of DMN was associated with an improvement in recall performance in patients with AD. Qi et al. (13) found remarkable damages to the DMN subsystems in patients with AD. Thus, the disruption of DMN is a characteristic change in AD that is related to AD symptoms (14). ECN is a task-positive network that is believed to be relevant to executive dysfunction. Zhao et al. observed changes in FC in patients with AD. They found that FC of the superior frontal gyrus, left thalamus, and middle frontal gyrus in ECN was significantly increased in patients with AD, suggesting the involvement of ECN disruption in AD (15, 16). SN is a task-positive network that is associated with attention, interoceptive awareness, and affection (17). Zhou et al. (18) reported decreased FC in DMN and increased FC in SN in AD subjects. Agosta et al. (14) reported increased FC in ECN and SN. Importantly, numerous studies have documented the key role of SN under AD conditions; it plays a “switching” role in the modulation of SN and ECN (17, 19–21). Damage to SN may affect other networks and eventually cause cognitive decline (21). In addition to these three networks, the frontoparietal network is reportedly involved in AD, in interacts with DMN, ECN, and SN (15, 16). Finally, changes in and interactions of these networks contribute to cognitive impairment in AD subjects.

However, there is insufficient evidence supporting the use of rs-fMRI in the diagnosis of aMCI. Hojjati et al. (22) reported a method that integrates rs-fMRI and structural magnetic resonance imaging (MRI) to predict the likelihood of aMCI becoming AD. Badhwar et al. (23) systematically reviewed 34 studies involving 1,363 patients in whom rs-fMRI was used to diagnose AD and aMCI. They suggested that resting-state connectivity can be considered a potential biomarker of AD. Zhang et al. (24) reported that abnormal FC was discovered in resting-state networks accompanying the progression of aMCI to AD. Such abnormal intra- and internetwork dysfunctions are potential biomarkers of aMCI progression. Recently, Ma et al. (9) have reported a typical disruption of DMN in AD and MCI subjects. However, studies investigating the direct relationship between aMCI and DMN, ECN, and SN are limited. Only two studies have simultaneously investigated abnormalities in all three networks in patients with aMCI. Early in 2012, Liang et al. (25) used seed-based correlation analysis (SCA) to investigate changes in FC in brain regions related to these three subsystems; they found that the FC of the angular gyrus related to DMN was significantly reduced along with an increase in the FC of the left frontal regions within ECN and the FC of some subcortical areas within SN. Later, Liao et al. (26) used the voxel-mirrored homotopic connectivity (VMHC) approach to evaluate the effects of changes in FC on DMN, ECN, and SN in aMCI subjects. On comparing the difference between AD and aMCI subjects, they determined that DMN inhibition and SN and ECN activation were the characteristic changes in patients with AD and aMCI. Moreover, VMHC was suggested to be a sensitive biomarker of AD as well as of the progression of aMCI to AD (26). On the basis of these findings, we believe that changes in DMN, ECN, and SN are important features in the progression of aMCI.

Independent component analysis (ICA) is a new method that separates signals of interest from noise in FC analysis (27). In contrast to SCA, ICA does not require a priori selection of a voxel, cluster, and region. It is always used to identify spontaneous activity patterns (27). Despite the strengths and weaknesses of ICA (28), in the present study, we attempted to explore changes in FC in brain regions attributed to DMN, ECN, and SN in patients with aMCI using ICA approach, which is different from SCA and VMHC approaches used in a previous study (28). We wanted to confirm whether changes previously reported in the aMCI state can be reproduced using the ICA approach. We believe that this study will strengthen the evidence for some characteristic changes in patients with aMCI detected using rs-fMRI and provide evidence that rs-fMRI is a useful tool for the early diagnosis of aMCI.

## Materials and Methods

### Participants

Patients with aMCI seen in our hospital between June 2018 and October 2019 were enrolled in this study. Healthy volunteers [the healthy control (HC) group] were also recruited. The study was conducted in strict compliance with the guidelines of the Declaration of Helsinki of the World Medical Association (2000) and was approved and supervised by the ethics committee of the First Affiliated Hospital of the Heilongjiang University of Chinese Medicine (approval number: HZYLLBA201910). All participants signed the informed consent form after the study protocol was explained to them in detail.

### Inclusion Criteria for the aMCI and Healthy Control Groups

We used the diagnostic criteria of the Chinese Dementia and Cognitive Impairment Diagnosis and Treatment Guidelines (2018) (29). Patients meeting the following criteria were included in the aMCI group: (1) those with cognitive impairment, which refers to memory impairment and other cognitive domain damage reported by the patient or the informed person and confirmed via objective examination; (2) those whose basic daily abilities were not affected and whose complex instrumental daily ability was only slightly damaged; (3) those who did not meet the diagnostic criteria for dementia; and (4) those with a geriatric depression scale score of 2–3, a memory test score 1.5 times lower than the standard deviation of the control group matched by age and education, a mini-mental state examination (MMSE) score ≥ 24, and a Hachinski Ischemic Score (HIS) ≤ 4. These criteria were consistent with Petersen's criteria (5).

The inclusion criteria for the control group were as follows: (1) no complaints of cognitive impairment; (2) no abnormalities detected on neurological examination and no visual or auditory impairment; (3) MMSE ≥ 27 points and multidimensional neuropsychological evaluation results within the corresponding normal range; and (4) no infarction or focal lesions on a conventional MRI scan.

The exclusion criteria for all subjects were as follows: (1) a history of nervous system or mental disorder (such as stroke, brain injury, brain tumor, anxiety, and depression); (2) incomplete demographic data; (3) inability to complete all neuropsychological tests; (4) inability to undergo image artifact or MRI examination; and (5) scanning head motion parameters of translation > 2 mm, rotation > 2°.

The cognitive state of all subjects was graded with a battery of neuropsychological assessments including the MMSE, Montreal Cognitive Assessment (MoCA), Trail Making Test (TMT), Digital Span Test (DST), Verbal Fluency Test (VFT), and Auditory Verbal Learning Test (AVLT); these were performed by an experienced neurologist who was blinded to the allocation of subjects and MRI analysis. The ADL scale, Hamilton Depression Rating Scale (HAMD), HIS, Global Deterioration Scale (GDS), and Clinical Dementia Rating (CDR) were also used for assessment.

### MRI Acquisition

A Philips 3.0T MR scanner (Achieva 3.0T-TX with dual gradient multisource emission; Philips Healthcare, the Netherlands) was used in the present study. All participants were asked to arrive 30 min before their test. They were asked to be fully rested to eliminate psychological factors such as fear, anxiety, and expectation. To reduce possible involuntary head movements caused by respiration and heartbeat, sponge pads were inserted on both sides of the head in the eight-channel parallel acquisition head coil (SENSE-N-8) for fixation.

First, all subjects were scanned using the conventional sequence, including T1WI, T2WI, and fluid-attenuated inversion recovery to exclude subjects who did not meet the inclusion criteria. Then, three-dimensional (3D) structural imaging data acquisition (T1W-3D-TFE sequence) was performed; the scanning parameters were as follows: time of repeat (TR) = 8.3 ms, time of echo (TE) = 3.8 ms, field of view (FOV) = 256 × 256 mm, flip angle (FA) = 12°, slices = 188, slice thickness = 1 mm, slice gap = 0 mm, and total scan duration = 4 min 52 s. Finally, functional imaging data (FE-EPI sequence) were collected using the following scanning parameters: TR = 2000 ms, TE = 30 ms, FOV = 220 × 220 mm, FA = 90°, matrix = 64 × 64, slices = 36, slice thickness = 3 mm, slice gap = 1 mm, total scan duration = 8 min 6 s, and a total of 240 time points.

### Data Analysis

Based on a MATLAB platform (R2017a; The MathWorks Inc., Natick, MA, USA), a DPARSF (version 4.3, http://rfmri.org/DPARSF) software package was run for image preprocessing. This process involved (1) converting data from the Digital Imaging and Communications in Medicine format to the NIfTI format; (2) removing the data of the first 10 time points to avoid the unstable interference of the MRI signal at the beginning of acquisition; (3) slice timing to ensure that all voxel acquisition times in a volume were consistent in theory; (4) realignment, the correction of small head movements between every volume of the subject (if the head translation was more than 2 mm and the rotation was more than 2°, the data for the subject were excluded; in this study, two patients with aMCI and one patient with normal cognitive function had head movement parameters beyond this range, and they were therefore excluded); (5) normalization, alignment of the local spatial data of different subjects to the Montreal Neurological Institute standard space and their resampling into 3 × 3 × 3 mm voxels; and (6) spatial smoothing, which is performed to reduce the registration error and increase the normality of the data for statistical analysis. This was done with the Gaussian kernel as the full width at half maximum 8 × 8 × 8 mm.

### Calculation of the Independent Components

ICA is a data-driven calculation method to separate functional components. It does not need a priori hypothesis, and the functional network components obtained are more realistic (30). Here we used methods introduced in a previous study for data processing (31). Briefly, ICA was performed using GIFT v4.0b software (Group ICA of fMRI toolbox, http://icatb.sourceforge.net). First, data were imported, the number of components was estimated using the software (30 in this study), and the dimensionality reduction was defined automatically (based on the levels of individuals and groups) using principal component analysis. The infomax algorithm was used. We selected the ICASSO method using both the “RandInit” and the “Bootstrap” options. The number of operations was 100. The minimum cluster size was 80, and the maximum cluster size was 100. Then, a spatial–temporal regression was used for the back-reconstruction algorithm, and the spatial components, time series of individuals, and groups were reconstructed. The spatial components were standardized to estimate the Z-scores, and then the average Z-score across the whole cluster was calculated. Based on information from a prior study (26) and a template provided by GIFT software, it was decided that DMN, ECN, and SN components would be acquired and analyzed statistically. Finally, for each subject, the chosen DMN, ECN, and SN components were converted to Z-values that represented the FC strength of DMN, ECN, and SN. Here we used the term “FC” to represent the increase or decrease in the statistical relationship among DMN, ECN, and SN in the resting-state networks. However, the analysis was not performed using the functional connectivity analysis approach.

### Statistical Analysis

SPSS software (v 25.0.0., IBM, IL, USA) was used for statistical analyses. Measurement data were presented as the mean ± standard deviation, and non-normally distributed data were presented as median (quartile). Chi-square test was used for comparing the classified variables between the two groups, and an independent-sample *t*-test was used for measurement data. Spearman rank correlation analyses were run to determine correlation between the Z-values of the functional connection and the MMSE scores of the two groups with significant differences in the three networks. Gender, age, and years of education were used as covariates for regression.

Statistical parametric mapping (SPM)-12 software (http://www.fil.ion.ucl.ac.uk/spm/) was used for statistical analyses of imaging indicators. In the control and aMCI groups, a single-sample *t*-test (family-wise error [FWE] correction, *P* < 0.05 as the test level) was used in the three network templates, and a combined template of the two single-sample brain FC areas was made using xjview (version 9.6, http://www.alivelearn.Net/xjview) software. Then, SPM12 software was used to run the *t*-test for two groups of the independent samples. The union template was used as the mask. Age, gender, and years of education were used as covariates. The statistical threshold was as follows: voxel level, *P* = 0.001 (uncorrected); cluster level, *P* < 0.05 (FWE-corrected); and cluster voxel size, >50. Thus, a *t*-value map with a significant between-group difference was obtained, and the results were displayed by xjview software.

## Results

Thirty patients with aMCI (13 males and 17 females) and 30 healthy subjects (14 males and 16 females) were enrolled in the study. All the subjects were right-handed. Table 1 shows the clinical characteristics of the subjects. There were no between-group differences in age, gender, and years of education. The results of a series of neuropsychological assessments, summarized in Table 1, showed that the aMCI group exhibited a significantly worse performance than the HC group (*P* < 0.01). Regarding clinical assessments, no significant between-group differences were observed in ADL and HIS (*P* > 0.05). However, the aMCI group exhibited higher HAMD, CDR, and GDS scores (indicating a poor performance) than the HC group (*P* < 0.01). The diagnoses of these patients were confirmed on the basis of the above findings (Table 1).

**Table 1:** Clinical characteristics of the enrolled subjects.

	**Items**	**HC**	**aMCI**	***P*-value**
Demographic characteristic	Number	30	30	−
	Age (y)	68.67 ± 3.19	68.53 ± 2.97	0.868
	Gender (M/F)	14/16	13/17	0.795
	Education (y)	9.93 ± 2.69	9.53 ± 2.64	0.563
Neuropsychological assessments	MMSE scores	28.20 ± 0.92	25.10 ± 0.66	0.000^**^
	MoCA scores	27.00 (1.00)	23.00 (1.00)	0.000^**^
	TMT-A (s)	56.28 ± 6.55	61.50 ± 6.14	0.012^**^
	TMT-B (s)	137.57 ± 10.03	180.10 ± 8.21	0.000^**^
	DST-Forward (min)	5.00 (1.00)	4.00 (1.00)	0.006^**^
	DST-Backword (min)	6.00 (1.00)	4.00 (0.75)	0.000^**^
	VFT (min)	20.81 ± 2.71	18.10 ± 2.29	0.001^**^
	AVLT- immediate recall (min)	6.95 ± 1.02	6.05 ± 0.89	0.005^**^
	AVLT-delayed recall (min)	6.52 ± 0.60	5.30 ± 0.47	0.000^**^
	AVLT- recognition (min)	9.90 ± 0.62	8.85 ± 1.18	0.001^**^
Clinical assessments	ADL	100.00 (0.00)	100.00 (0.00)	0.069
	HAMD	5.00 (0.00)	6.00 (1.00)	0.004^**^
	HIS	3.00 (1.00)	3.00 (2.00)	0.428
	GDS	1.00 (0.00)	3.00 (0.75)	0.000^**^
	CDR	0.00 (0.00)	0.5 (0.00)	0.000^**^

*ADL, Activity of Daily Living Scale; aMCI, amnestic mild cognitive impairment; AVLT, Auditory Verbal Learning Test; CDR, Clinical Dementia Rating; DST, Digital Span Test; GDS, Global Deterioration Scale; HAMD, Hamilton Depression Scale; HC, healthy control; HIS, Hachinski Ischemic Scale; MMSE, Mini-Mental State Examination; MoCA, Montreal Cognitive Assessment; TMT, Trail Making Test; VFT, Verbal Fluency Test*.

*Date of normal distribution are presented as the mean ± SD, Date of non-normal distribution are presented as the median (quartile)*,

** *means p < 0.01*.

**Figure 1:**
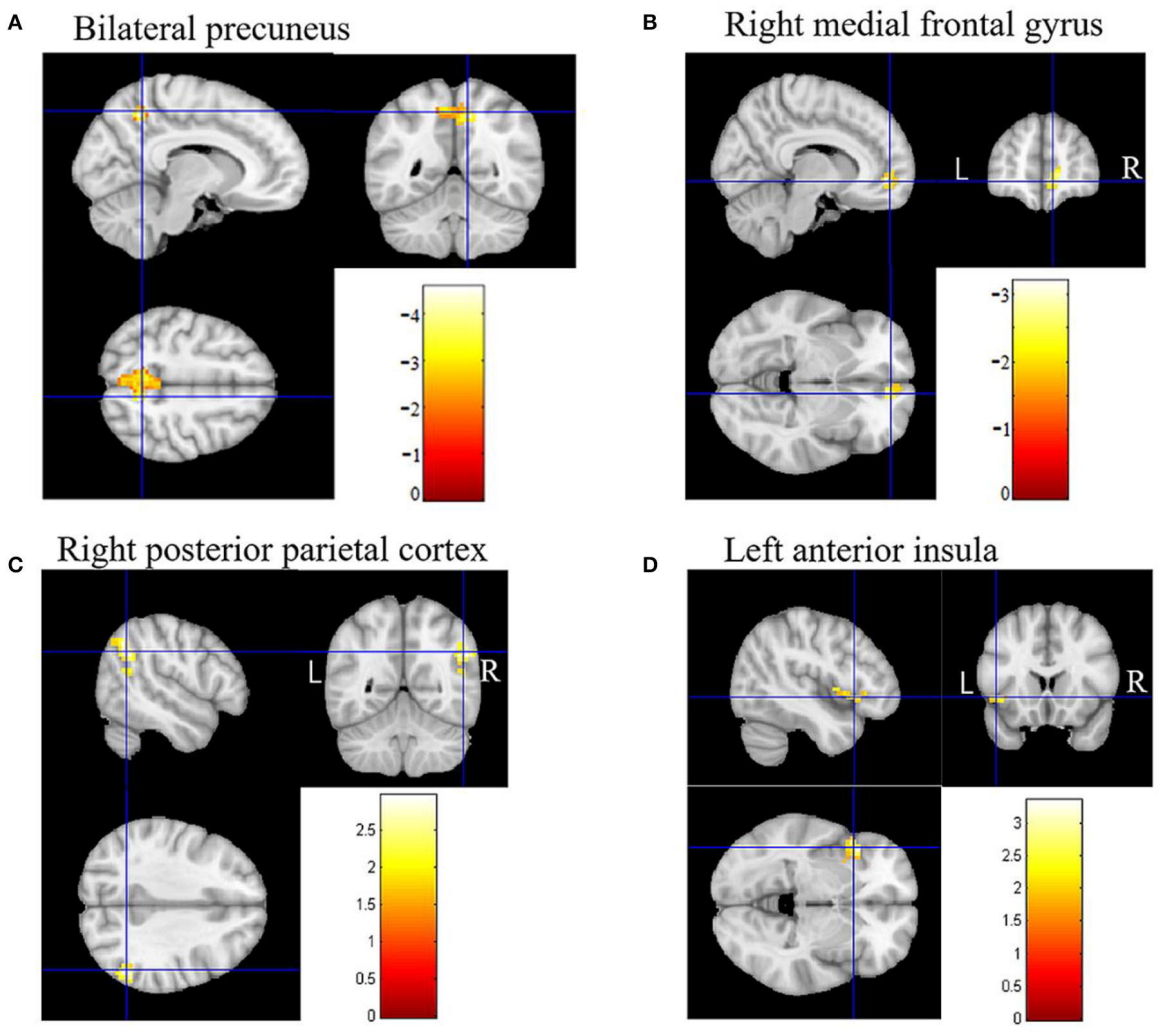
Brain regions of functional connectivity (FC) that were changed in patients with amnestic mild cognitive impairment (aMCI). **(A)** The FC of the default mode network (DMN) of the bilateral anterior precuneus in patients with aMCI decreased. **(B)** The FC of DMN of the right medial frontal gyrus in patients with aMCI decreased. **(C)** The FC of the executive control network (ECN) of the right posterior parietal cortex in patients with aMCI increased. **(D)** The FC of the salience network (SN) of the left anterior insula in patients with aMCI increased.

**Figure 2:**
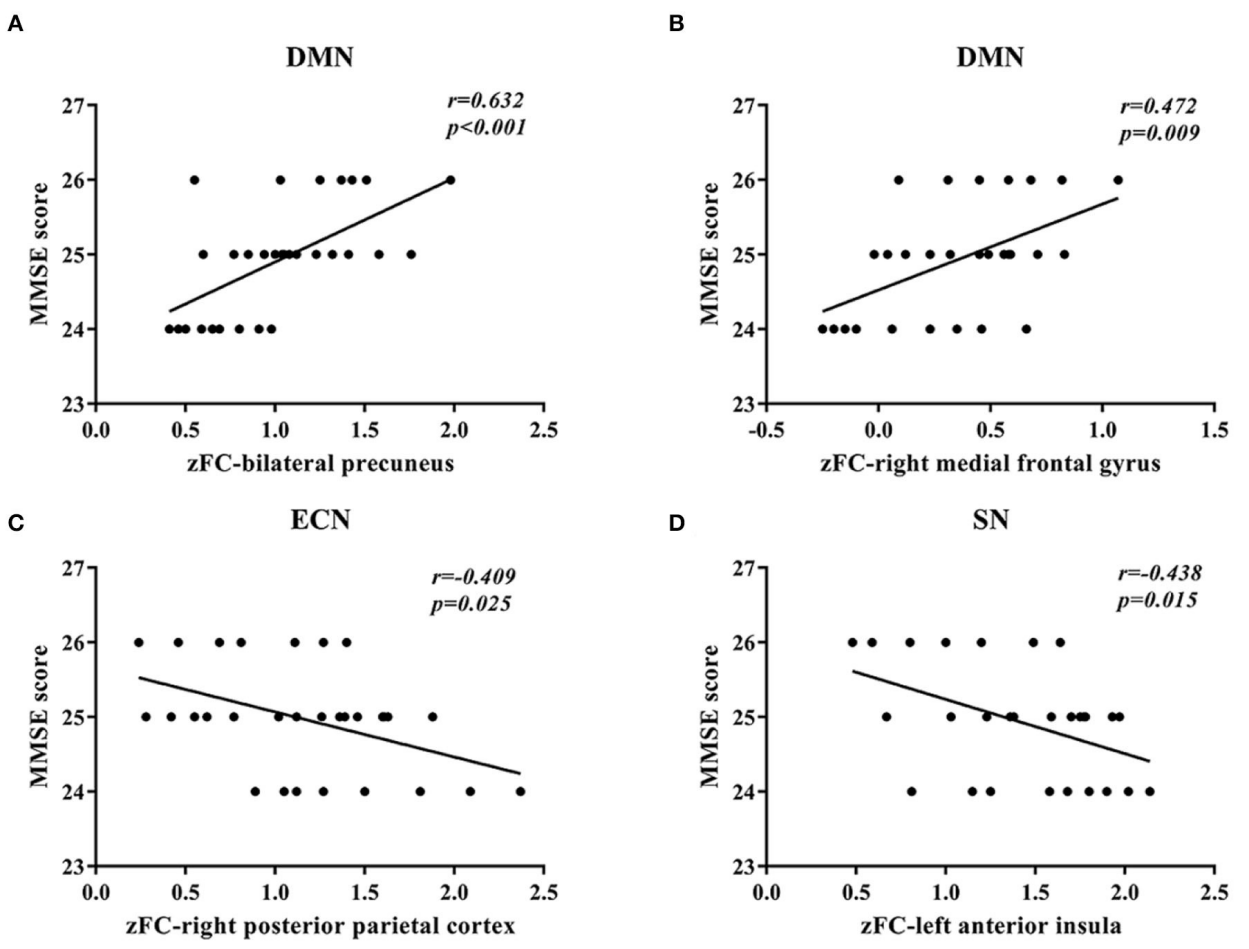
Correlation between the Z-values of brain regions and the mini-mental state examination (MMSE) scores. **(A)** A significant positive correlation between the Z-value of the default mode network (DMN) of the bilateral anterior precuneus and the MMSE scores was found in patients with amnestic mild cognitive impairment (aMCI). **(B)** A significant positive correlation between the Z-value of the default mode network (DMN) of the right medial frontal gyrus and the MMSE scores was found in patients with aMCI. **(C)** A significant negative correlation between the Z-value of the executive control network (ECN) of the right posterior parietal cortex and the MMSE scores was found in patients with aMCI. **(D)** A significant negative correlation between the Z-value of the salience network (SN) of the left anterior insula and the MMSE scores was found in patients with aMCI.

In DMN, the FC of the bilateral anterior precuneus (Figure 1A) and right medial frontal gyrus (Figure 1B) was decreased significantly in the patients with aMCI compared with that in the HCs. No significant increase was found in the DMN detected in the present study (Table 2). These results were confirmed via the related analysis. Significant positive correlations were found between the Z-values of the bilateral anterior precuneus and the MMSE scores (*r* = 0.632; *P* < 0.001; Figure 2A) and between the Z-values of the right medial frontal gyrus and the MMSE scores (*r* = 0.472; *P* = 0.009; Figure 2B).

**Table 2:** Functional connectivity (FC) in the three networks [patients with amnestic mild cognitive impairment (aMCI) vs. healthy controls (HCs)].

**Networks**	**Cluster size**	**Brain regions**	**Brodmann**	***t*-value**	**MNI**		
					**x**	**y**	**z**
DMN	53	Right medial frontal gyrus	BA10	−3.2006	9	48	−6
	159	Bilateral anterior precuneus	BA7	−4.5941	12	−54	48
ECN	96	Right posterior parietal cortex	BA40	2.9443	54	−54	33
SN	50	Left anterior insula		3.3464	−42	21	−3

*In the default mode network (DMN), t-value = −3.2006 and −4.5941, which means that FC in the aMCI group decreased compared with that in the HC group*.

*In the executive control network (ECN), t-value = 2.94443, which means that FC in the aMCI group increased compared with that in the HC group*.

*In the salience network (SN), t-value = 3.3464, which means that FC in the aMCI group increased compared with that in the HC group*.

*Voxel-level initial threshold, P ≤ 0. 001 (uncorrected)*.

*Cluster-level threshold, P < 0.05 [family-wise error (FEW) correction]*.

In ECN, the FC of the right posterior parietal cortex (Figure 1C) was increased significantly in the patients with aMCI compared with that in the HCs. No significant decrease was found in the ECN detected in the present study (Table 2). These results were confirmed via the related analysis. Significant negative correlations were found between the Z-values of the right posterior parietal cortex and the MMSE scores (*r* = −0.409; *P* = 0.025; Figure 2C).

In SN, the FC of the left anterior insula (Figure 1D) was increased significantly in patients with aMCI compared with that in HCs. No significant decrease was found in the SN detected in the present study (Table 2). These results were confirmed via the related analysis. Significant negative correlations were found between the Z-values of the left anterior insula and the MMSE scores (*r* = −0.438; *P* = 0.015; Figure 2D).

**Figure 3:**
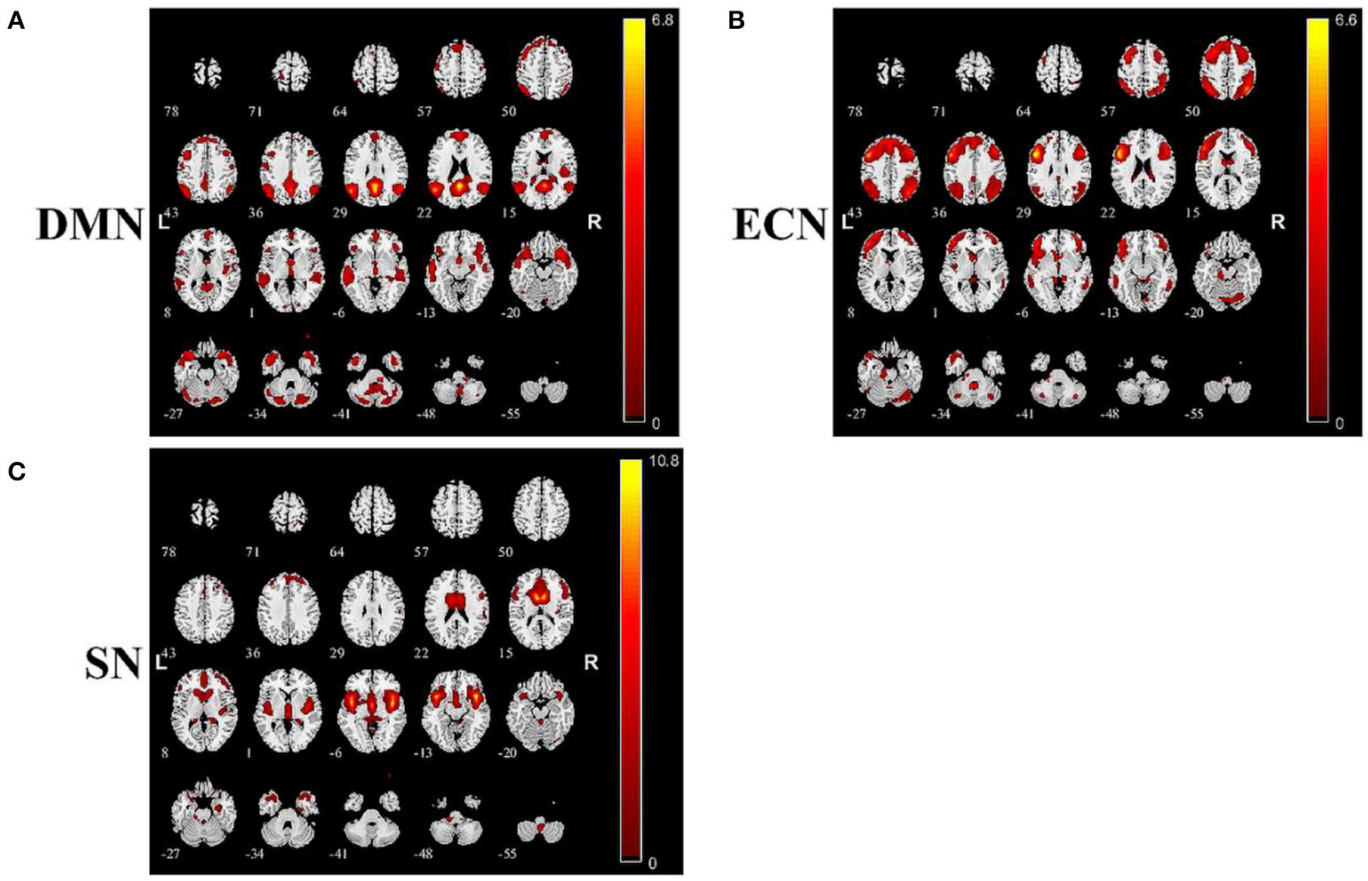
Summary of images of brain function networks detected in this study: **(A)** default mode network (DMN), **(B)** executive control network (ECN), and **(C)** salience network (SN).

The anatomical model of DMN, ECN, and SN detected using ICA approach in the present study are summarized in Figure 3.

## Discussion

In the present study, we investigated changes in the cerebral FC in DMN, ECN, and SN in patients with aMCI using rs-fMRI. First, we found that the significant decrease in FC in brain regions can be attributed to DMN and the increase in FC in brain regions can be attributed to ECN; these findings are in accordance with previous studies on AD and MCI. This confirmed the reliability of our experimental system. Second, the most important finding in this study was that the increase in FC in brain regions can be attributed to SN, which is different from the previous findings in patients with AD. These results might reflect the pathophysiological state of aMCI, which requires further confirmation by setting up an AD group in the future investigation. The results are in agreement with those of previous studies using the SCA (25) and VMHC (26) approaches. We therefore considered that the decrease in FC in DMN and the increase in FC in ECN and SN are peculiar patterns observed on rs-MRI of a person with aMCI. To our knowledge, this is the first report to simultaneously investigate the changes in three core cerebral networks in patients with aMCI using the ICA approach. We believe that these findings will be helpful in developing an imaging biomarker for the diagnosis of aMCI, which is crucial for ameliorating the clinical outcome and improving quality of life (QOL) of patients with dementia.

Our clinical and neuropsychological examination findings confirmed the diagnosis of the patients with aMCI. The MMSE and MoCA scores; TMT and DST results in both directions; and VFT results, GDS and HAMD scores, and AVLT results (immediate, delayed recalls, and recognition) indicated a worse state in the aMCI group. However, there were no between-group differences in ADL and HIS. These findings are consistent with the diagnostic criteria used in the present study (29) as well as with Petersen's criteria (5) (Table 1).

### Findings in Accordance With Previous Studies on AD and aMCI

We found that the FC of the bilateral anterior precuneus and right medial frontalis gyrus, which belong to DMN, was reduced significantly in the aMCI group than in the HCs group (Figure 1B). The subsequent related analyses confirmed these findings. The Z-values of the bilateral anterior precuneus and right medial frontal gyrus were positively correlated with the MMSE scores (Figures 2A,B). Lower MMSE scores were related to lower Z-values, which means that worse cognition was related to lower connectivity. These findings are in agreement with those of the following previous studies. Qi et al. (32) found decreased FC in the hippocampal region, bilateral anterior precuneus, posterior cingulate gyrus, right inferior parietal lobule, and left fusiform gyrus in patients with aMCI. Damoiseaux et al. (33) reported a reduction in FC in the posterior junction of DMN in patients with mild AD, which was detected using ICA. Pappas et al. (34) found that the MMSE scores were correlated with choline acetyltransferase (ChAT) activity in the medial frontal cortex in patients with AD. Ikonomovic et al. (35) reported that lower MMSE scores were related to a reduced precuneus ChAT activity.

DMN is the most basic resting-state network, and it plays a role in integrated primary perception, advanced cognitive function, situational memory extraction, self-consciousness, and cognitive processes. It is also closely related to social cognition, spatial perception, and other brain functions (36). Anatomically, DMN includes mainly the posterior cingulate gyrus, anterior precuneus, medial prefrontal cortex, superior frontalis, middle frontalis, inferior parietal lobule, middle temporal gyrus, infratemporal gyrus, and hippocampus (37). Our findings are in agreement with the results of previous studies on patients with aMCI (32) and AD (33, 38, 39); these studies have reported that in these patients, the FC of DMN is decreased, indicating damage to FC in the state of cognitive impairment.

We found that the FC of the right posterior parietal cortex in ECN was increased in the aMCI group than in the HC group (Figure 1C), which was confirmed via the related analysis, namely, the Z-value of the right posterior parietal cortex was negatively correlated with the MMSE scores (Figure 2C). These findings are in accordance with those of previous analogous studies on patients with aMCI (40) and mild AD (41). ECN includes the dorsolateral prefrontal cortex, cingulate gyrus in the dorsolateral prefrontal cortex, and posterior parietal cortex (42). ECN is involved in multiple high-level cognitive tasks, which are crucial for actively maintaining and manipulating information in working memory, rule-based problem-solving, and goal-based behavior decision-making (43). Our findings suggest that the increase in the right posterior parietal cortex might be a compensation effect of the decline in DMN FC. This indicates an antagonistic and complementary relationship between DMN and ECN, which is consistent with the findings of a previous study (44). These findings replicate those of previous studies on patients with AD and, importantly, on patients with aMCI, proving that our experimental design and system were reliable.

### New Findings Concerning Changes in FC in Brain Regions Related to aMCI

Importantly, we found that the FC of the left anterior insula in SN was increased significantly in the aMCI group than in the HC group (Figure 1D), which was confirmed via the related analysis, namely, the Z-value of the left anterior insula was negatively correlated with the MMSE scores (Figure 2D). These findings are contrary to previous results from patients with AD. Data from patients with AD indicate that FC in SN was decreased, indicating damage to FC in these patients (18, 45). There has been no analogous study regarding these changes in patients with aMCI. SN comprises a bilateral forebrain island and an anterior cingulate gyrus. Like ECN, it is an active mission network, participating in brain directional information processing, evaluating and selecting the most relevant environmental stimuli, classifying different stimuli and events, and switching between related processing systems (46). The anterior insula, which is located on the forebrain island, is a key node of SN, which is connected to multiple areas of the prefrontal cortex, central anterolateral cortex, parietal lobe, and temporal lobe (47). It plays an important role in attention and transformation between cognitive domains (48). On the basis of this knowledge, it is easy to understand that in the AD state, the functional activity of SN is reduced because the brain areas connected to SN are also decreased. However, we found that SN was activated in the aMCI state, which is different from the results found in patients with AD. This might be due to differences between the pathophysiological states of patients with AD and aMCI. Our data did not support that there was damage to FC in SN in the aMCI stage. One explanation for this is that the increase in FC in the forebrain island might be due to anxiety and depression in aMCI. Another explanation is that the increase in FC in the forebrain island is a compensatory effect for the damage to FC in DMN.

Interestingly, previous studies using the SCA and VMHC approaches have obtained similar results, that is, a decrease in DMN and an increase in ECN and SN in patients with aMCI (25, 26). We, therefore, believe that changes in DMN, ECN, and SN comprise a pattern that is peculiar to the resting-state network in patients with aMCI. However, Liao et al. (26) found that patients with AD also exhibited this pattern. This result is different from those of other studies on AD that suggested a decrease in SN (18, 45). This difference might be associated with a different experimental design (AD vs. aMCI, without normal control) and, in particular, with different VMHC approaches, which require further investigation.

The relationship among these three networks is complicated and multidimensional and is not fully understood. Normal function with flexible interactions among DMN, ECN, and SN plays a vital role in the maintenance of normal social behavior (49). These networks are usually coadjusted according to different physiological states. For example, during performance of a working memory task, the networks changed with the increase in task load to maintain the best performance. FC within DMN was reduced and that within ECN was increased. The difference in FC between SN and DMN as well as that between SN and ECN remarkably increased (50). In terms of the association among these networks, the key switching role of SN in the modulation of DMN and ECN has been well-documented (17, 19–21). The right anterior insula in SN plays a key role in modulating ECN and DMN for better task performance (51). An intact SN drives DMN and ECN during resting and task states, respectively, and contributes to maintaining normal cognitive function in healthy young (52) and old (21) subjects. Nevertheless, the interaction among the three networks in the AD or aMCI state remains to be fully understood. Chand et al. (21) found that patients with MCI exhibited damaged interactions among DMN, ECN, and SN. The SN-centered control model was impaired, resulting in a changed modulation pattern of the networks. Later, Li's study confirmed these changes. Internetwork connectivity, especially SN–DMN and SN–ECN, is increased in patients with AD and late MCI, suggesting an abnormal state of SN (20). These findings are in agreement with those of the present study. Some authors believe that the changes in FC observed in patients with aMCI are compensatory effects (53). Yi et al. (54) reported that DMN is the most important network to distinguish between aMCI with and without amyloid-beta (Aβ) protein deposition. A reciprocal variability balance between DMN and SN was observed in aMCI (55). The findings of the present and previous studies imply that atypical activation of SN and subsequent activation of ECN are the compensatory mechanisms responsible for the decreased function of DMN under MCI conditions, in which SN has to obtain more resources to maintain brain function. This hypothesis based on the compensatory mechanisms of the complicated interactions among SN, DMN, and ECN under the condition of aMCI or AD needs verification in our future investigation.

There are several limitations to this study. First, our study lacked an AD group; this was the main limitation of our study. We only performed comparisons between patients with aMCI and HCs. From the results of the present study, we cannot obtain direct evidence concerning the difference between AD and aMCI. Moreover, although we found the depression scores in aMCI group were significantly higher than those of HC group, we don't know the depression state in AD patients. Thus, the potential confounding role of the depression in the cognitive impairment is uncertain in the present experimental system. Hence, adding patients with AD to these comparisons would be more meaningful. In the future, we will set up an AD group to perform a direct comparison that will permit a stronger conclusion. Second, we did not perform more neuropsychological assessments for testing the non-memory domains to demonstrate that there was no non-memory impairment in the included patients. Although it has been reported that patients with aMCI may suffer from other impairments as well, such as impairment of executive function (56), the included patients might be confused with patients with multiple-domain MCI. This may lead to heterogeneity among the included patients (57). Third, the enrolled patients were diagnosed on the basis of clinical examination findings. We did not confirm the diagnoses using a biomarker for Aβ. Fourth, similar to other approaches, the ICA approach used in this study has several limitations (28). ICA is a data-driven approach to separate functional components during signal processing. It is based on a blind source separation algorithm and does not need an a priori hypothesis as mentioned before. Hence, it can be used for analysis where a detailed a priori model is not available. However, some challenges remain for the ICA approach: (1) There are no a priori criteria to identify the number of independent components in blood oxygenation level-dependent data, which contribute to the final results to a high degree. (2) There is run-to-run variability; data obtained from different runs of ICA, even on the same data, might be variable. (3) The results of ICA might be split into many subnetworks. Thus, numerous components, which are difficult to identify, would have to be estimated (28). Fifth, these networks are defined by their connectivity, rather than by their constituent structures (10). Although the correlations between the functional networks and the constituent structures are not fully understood, it has been verified that gray matter atrophy may influence the functional results (10, 25, 58). Thus, gray matter atrophy must be seriously considered in functional imaging studies of neurodegenerative diseases (25, 58). However, the present study did not control for gray matter atrophy. Thus, the reported FC changes may have been confused with the effects of gray matter atrophy in the patients with aMCI. All these limitations reduce the reliability of the evidence and should be addressed in our future studies.

Taken together, we found that in the aMCI state, the FC of brain areas in DMN was decreased, whereas that in ECN was increased, which is in agreement with the changes seen in patients with AD. However, FC in SN was also increased in patients with aMCI, which was contrary to the findings in patients with AD, indicating that damage to FC in SN does not occur in the aMCI stage; this could mean that there are differences in the pathophysiological states between AD and aMCI. Compensatory response may occur in the aMCI state but seldom in the AD state (53). Moreover, these results are consistent with those obtained using different analytical approaches. Thus, a decrease in DMN and an increase in ECN and SN may be peculiar patterns observed on rs-fMRI of a person with aMCI and require further investigation.

## Conclusion

The FC of brain regions was decreased in DMN and increased in ECN and SN, which may be a peculiar pattern observed on rs-fMRI in the aMCI stage. These findings may contribute to developing imaging biomarkers for the diagnosis of aMCI; this would be further beneficial for ameliorating clinical outcomes and improving QOL of patients with dementia.

## Data Availability Statement

The raw data supporting the conclusions of this article will be made available by the authors, without undue reservation.

## Ethics Statement

The studies involving human participants were reviewed and approved by the ethics committee of the First Affiliated Hospital of the Heilongjiang University of Chinese Medicine (approval no: HZYLLBA201910). The patients/participants provided their written informed consent to participate in this study.

## Author Contributions

XL, FW, XL, TY, and TA developed the original idea and designed the approach. XL, FW, XL, DC, LC, XJ, XY, and TY contributed to acquisition, analysis, or interpretation of data for the work. XL, FW, XL, and TA wrote the first draft. TY and TA revised the manuscript critically for important intellectual content. TA and TY supervised the study. All authors approval of the final version to be submitted.

## Conflict of Interest

The authors declare that the research was conducted in the absence of any commercial or financial relationships that could be construed as a potential conflict of interest.
